# Characterization of METTL7B to Evaluate TME and Predict Prognosis by Integrative Analysis of Multi-Omics Data in Glioma

**DOI:** 10.3389/fmolb.2021.727481

**Published:** 2021-09-17

**Authors:** Xiaochuan Chen, Chao Li, Ying Li, Shihong Wu, Wei Liu, Ting Lin, Miaomiao Li, Youliang Weng, Wanzun Lin, Sufang Qiu

**Affiliations:** ^1^ Department of Radiation Oncology, Fujian Medical University Cancer Hospital, Fujian Cancer Hospital, Fuzhou, China; ^2^ Department of Oncology, Sanming Second Hospital, Sanming, China; ^3^ Department of Radiation Oncology, Shanghai Proton and Heavy Ion Center, Fudan University Cancer Hospital, Shanghai, China; ^4^ Fujian Provincial Key Laboratory of Translational Cancer Medicine, Fuzhou, China

**Keywords:** glioma, prognosis, immunosuppressive microenvironment, multi-omics, METTL7B

## Abstract

Glioma is the most common and aggressive type of primary brain malignant tumor with limited treatment approaches. Methyltransferase-like 7B (METTL7B) is associated with the pathogenesis of several diseases but is rarely studied in glioma. In this study, 1,493 glioma samples (data from our cohort, TCGA, and CGGA) expressing METTL7B were used to explore its prognostic value and mechanism in the immune microenvironment. Results showed that high expression of METTL7B is associated with poor prognosis and abundant immunosuppressive cells. Further, functional enrichment showed that METTL7B is involved in the negative regulation of immunity and carcinogenic signaling pathways. Moreover, a METTL7B-related prognostic signature constructed based on multi-omics showed a good prediction of the overall survival (OS) time of glioma patients. In conclusion, METTL7B is a potential prognostic biomarker. In addition, the prognostic prediction model constructed in this study can be used in clinical setups for the development of novel effective therapeutic strategies for glioma patients and improving overall survival.

## Introduction

Glioma is the most common and aggressive type of primary brain malignant tumor (Jiang et al., 2016; Lapointe et al., 2018). However, current therapy approaches, for glioma including surgery, chemotherapy, and radiotherapy, are not fully effective. Therefore, treatment of glioma is a challenge resulting in high mortality rates. WHO classification system defines diffuse low-grade glioma (LGG) as the WHO grade II/III based on histological type (Wesseling and Capper 2018). Most LGGs show recurrence and gradually transform into higher-grade gliomas (GBM) leading to death (Louis et al., 2007; Liu et al., 2019). The median survival time of GBM patients is approximately 14.6 months, and the 5-year survival rate is less than 10% (Stupp et al., 2005; Stupp et al., 2009). Therefore, novel effective biomarkers for the prediction of the prognosis of patients with glioma should be explored to improve clinical outcomes.

Human methyltransferase-like (MettL) proteins are involved in methylation reactions. Although members of this protein family play key biological functions, for instance, METTL2B, METTL3, METTL8, and METTL16 are RNA methyltransferases and are implicated in tumorigenesis, the roles of METTL proteins are not clear (Xu K et al., 2017; Xu L et al., 2017; Deng et al., 2018; Ignatova et al., 2019). Methyltransferase-like 7B (METTL7B), also known as associated with LD protein 1 (ALD1), is localized on chromosome 12. Studies report that METTL7B is implicated in diseases, such as infection Abdel-Hameed et al. (2014), non-alcoholic steatohepatitis lipid metabolism Thomas et al. (2013), and several tumors, which include primary thyroid cancer (PTC), lung adenocarcinoma (LUAD), and non-small cell lung cancer (NSCLC). In PTC, METTL7B is upregulated and promotes tumor invasion and malignancy by activating the TGF-β1-induced EMT (Cai et al., 2018). In LUAD, a recent study demonstrates that METTL7B is overexpressed in NSCLC tumor tissues and promotes tumorigenesis by regulating cell cycle progression (Ali et al., 2020). Moreover, METTL7B promotes tumorigenesis by regulating cell cycle progression in non-small cell lung cancer (Liu et al., 2020). However, the oncogenic role and prognostic value of METTL7B in glioma has not been reported previously.

This study aimed to elucidate the efficacy of METTL7B, as a potential diagnostic and prognostic biomarker for glioma. Expression levels of METTL7B mRNA in glioma tissues and normal brain tissues were determined. Further, survival analysis, independent prognostic analysis, ROC curve analysis, and clinical correlation analysis were used to determine the clinical and prognostic value of METTL7B. In addition, gene set enrichment analysis (GSEA) was used for functional and pathway analysis. Immune infiltration correlation analysis was performed to determine the role of METTL7B in the tumor immune microenvironment. Multi-omics data (WES and DNA methylation array) were then analyzed. Finally, a prognosis prediction model was constructed based on METTL7B-related significant alterations. The findings from this study form a basis for the development of an effective predictor for the prognosis of gliomas.

## Materials and Method

### Data Retrieval

Data were retrieved from the GEPIA database (http://gepia.cancer-pku.cn/index.html) (Tang et al., 2017). Expression data were used to explore differences in expression levels of METTL7B mRNA in glioma tissues and normal brain tissues. RNA expression data and corresponding clinical data of 666 and 693 glioma (LGG + GBM) patients were retrieved from the TCGA database using the UCSC website (https://xenabrowser.net/) (Kent et al.,2002) and from the CGGA website (http://www.cgga.org.cn/), respectively. In addition, somatic mutation data (VarScan2 Variant Aggregation) and DNA methylation data (the Illumina 450K methylation array) of glioma were retrieved from the TCGA database (https://portal.gdc.cancer.gov/).

A total of 134 glioma specimen tissues (G6042) were purchased from Servicebio Company (Wuhan, China) and used to further validate METTL7B expression and its prognostic value in glioma.

### Immunohistochemistry (IHC) Analysis

Glioma tissues sections were analyzed through IHC using anti-human METTL7B (Proteintech, Cat #17001-1-AP). HRP-linked secondary antibodies (Abcam, Cat #ab205718, UK) were then used followed by DAB treatment. Images were obtained under a microscope (3DHISTECH, Hungary) at ×20 magnification. Histochemistry score (H-score) was used to detect and quantify the expression level of METTL7B. H-score was calculated as follows: H-score = (percentage of cells of weak intensity × 1) + (percentage of cells of moderate intensity × 2) + (percentage of cells of strong intensity × 3).

### Single-Cell Level Analysis

We obtained GBM single-cell sequencing data (GSE131928, GSE139448, GSE84465) from the online database of the Tumor Immune Single-Cell Hub (TISCH) (http://tisch.comp-genomics.org/) (Neftel et al., 2019; Sun et al., 2021), which was used to classify malignant cells, immune cells, and stromal cells by hierarchical clustering. Then, the expression of METTL7B in these cells was evaluated, and the results were illustrated by heatmaps.

### Gene Set Enrichment Analysis

GSEA was used to compare expression levels between a priori defined set of genes and high and low METTL7B expression groups in the enrichment of MSigDB Collection (c2.cp.kegg and c5.go.bp. v7.2. symbols.gmt). High and low METTL7B were then used as a phenotype label and gene set permutations were carried out 1,000 times for each analysis. False discovery rate (FDR) and normalized enrichment score (NES) were used to classify gene ontology (GO) and KEGG pathways enriched in differential phenotype.

### DNA Methylation Analysis

Limma package in R was used to normalize the gene methylation matrix. The Pearson correlation coefficient was used to determine the association between the gene expression and DNA methylation level of METTL7B. The Kaplan–Meier curves of differential METTL7B DNA promoter CpG sites in glioma patients were constructed using the R survival package.

### Somatic Mutation Analysis

WES somatic mutations data of both high METTL7B (n = 327) and low METTL7B groups (n = 329) were used to detect the SNVs, SNPs, and INDELs using VarScan2.39 software. Differentially mutated genes, which were defined with a *p* value lower than 0.05, were analyzed using Fisher’s exact test. R maftools package was used for visualization of somatic mutations and calculation of TMB score.

### Analysis of Immune Infiltration

The abundance of immune cells was determined using CIBERSORT (22 immune cell types) and xCell (64 immune and stromal cell types) algorithms (Newman et al., 2015). Mann–Whitney *U* test was performed to compare differential immune cell distribution between high and low METTL7B expression groups. Further, the expression of genes which negatively regulate the Cancer-Immunity Cycle Chen and Mellman (2013) was determined in low and high METTL7B expression groups. Immunosuppressive gene signatures were retrieved from the Tracking Tumor Immunophenotype website (http://biocc.hrbmu.edu.cn/TIP/index.jsp) (Xu et al., 2018).

### Construction and Evaluation of Prognosis Prediction Model

Survival time and status were determined to assess the prognosis of glioma patients. Cases that included their overall survival were considered. Prognosis prediction models were constructed based on gene expression values of METTL7B, differential immune cells and immunosuppressive gene sets, gene mutation, and methylation probe signals. Cases with all characteristics being investigated were considered for the model with comprehensive characteristics from multi-omics data, and 545 same cases were obtained.

Genes were expressed as 0 (wild) and 1 (mutation) based on somatic mutation. Immune cell fraction level was expressed as 0 or 1 (not = 0). Univariate Cox regression analysis was performed to determine prognostic factors. Significant predictive features were further identified by LASSO-COX analysis. A prognosis signature was constructed based on coefficients from LASSO-COX analysis. SurvivalROC package in R was used to measure predicting ability of the prognosis model using the Kaplan–Meier survival curves and time-dependent receiver operating characteristic (ROC) curves. The R rms package was used to construct a prognostic nomogram based on the model and clinical information of glioma patients. Further, calibration plots for 3 and 5 years were constructed to validate predicted and actual probabilities.

### Statistical Analysis

Expression of METTL7B in tumor and normal tissues was estimated using Wilcoxon signed-rank test. OS of participants were compared between high and low METTL7B expression groups through Kaplan–Meier analysis using the R Survival and Survminer package. Univariate Cox analysis was used to determine potential prognostic factors, whereas multivariate Cox analysis was performed to identify METTL7B expression as an independent risk factor for OS in glioma patients. The receiver operating characteristic (ROC) curve was used to evaluate the diagnostic value of METTL7B expression using the R survivalROC package. The area under curve (AUC) represented the diagnostic value. Correction between clinical pathologic features and METTL7B expression was analyzed using Wilcoxon signed-rank test or Kruskal–Wallis test. *p* < 0.05 was considered statistically significant. All data analyses were carried out using R software (version 3.6.0) and AdobeIllustratorCS6.

## Results

### Clinical Prognostic Value of METTL7B Expression in TCGA and CGGA Database

**FIGURE 1:**
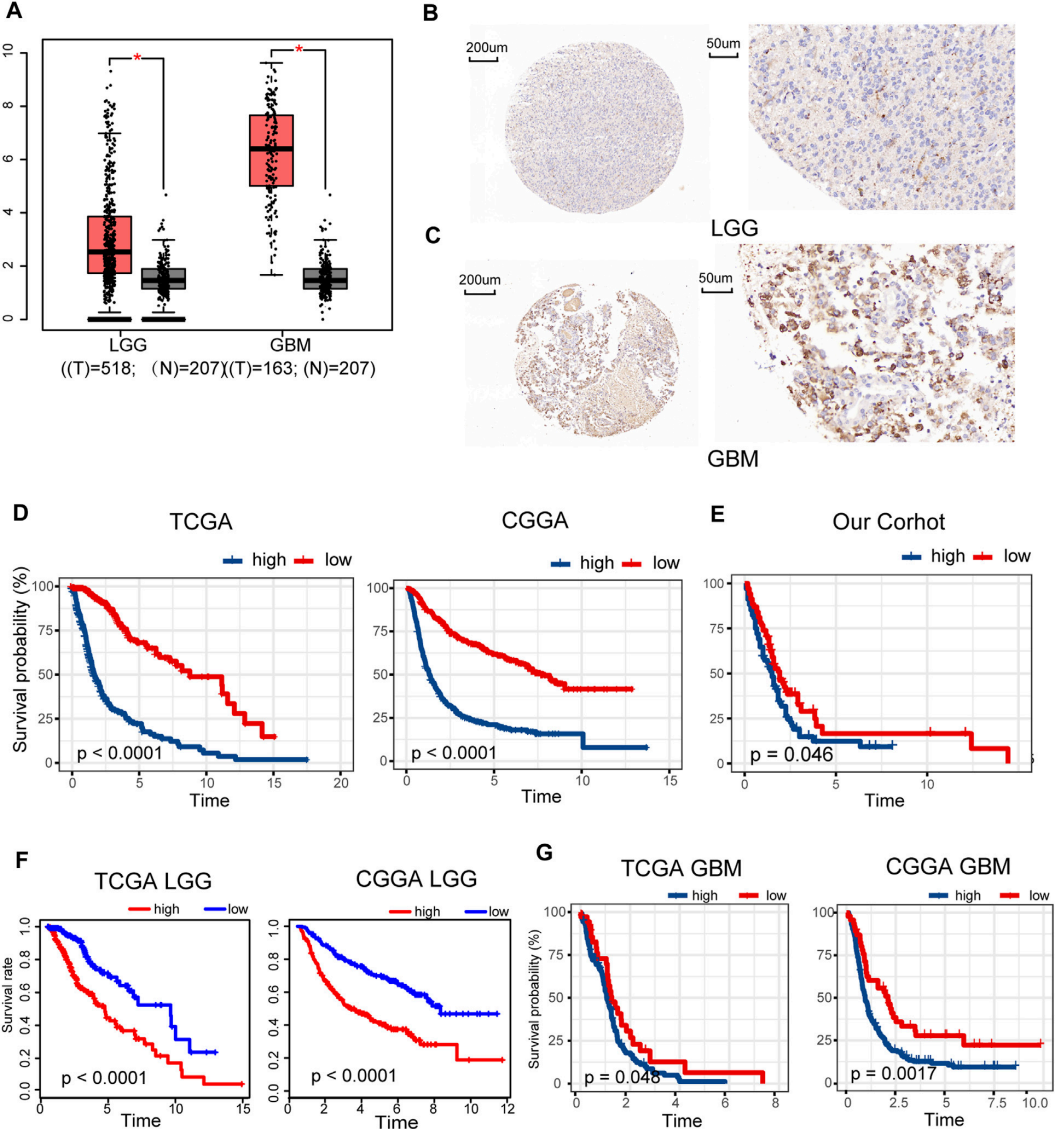
Clinical prognostic value of METTL7B expression in LGG and GBM patients. **(A)** METTL7B expression of normal brain tissues from GTEx data (n = 207) compared with expression in LGG (n = 518) and GBM (n = 163) samples retrieved from TCGA database. **(B–C)** Representative specimens exhibiting METTL7B IHC labeling pattern in LGG and GBM. **(D–E)** Survival analysis of patients with glioma in high METTL7B and low METTL7B groups in the TCGA, CGGA, and our cohort. **(F)** KM survival curve of patients with LGG in high METTL7B and low METTL7B groups in TCGA and CGGA datasets. **(G)** KM survival curve of patients with GBM in high METTL7B and low METTL7B groups in TCGA and CGGA datasets. *p < 0.05, **p < 0.01, ***p < 0.001, and ****p < 0.0001.

Gene expression data of 518 LGG and 163 GBM samples from TCGA and 207 normal brain tissues from GTEx portal were dissected using GEPIA. Expression levels of METTL7B mRNA were higher in LGG and GBM tissues, compared with the expression levels in normal tissues (Figure 1A). IHC staining data of LGG and GBM were used to evaluate the expression level of METTL7B in glioma tissues. Overall, positive staining for METTL7B was detected in GBM tissue, whereas LGG samples showed negative staining for METTL7B (Figures 1B, C). The Kaplan–Meier analysis of the TCGA and CGGA dataset (including GBM and LGG) showed that a high expression level of METTL7B was associated with poor prognosis (Figure 1D). This differential expression was validated using our dataset (Figure 1E). In addition, the prognostic value of METTL7B for different WHO grades was evaluated. LGG patients with high METTL7B expression levels showed significantly shorter OS time compared with patients with low METTL7B expression level in CGGA cohorts (Figure 1F). The High METTL7B expression level of GBM patients was associated with poor OS in TCGA and CGGA cohorts (Figure 1G).

**FIGURE 2:**
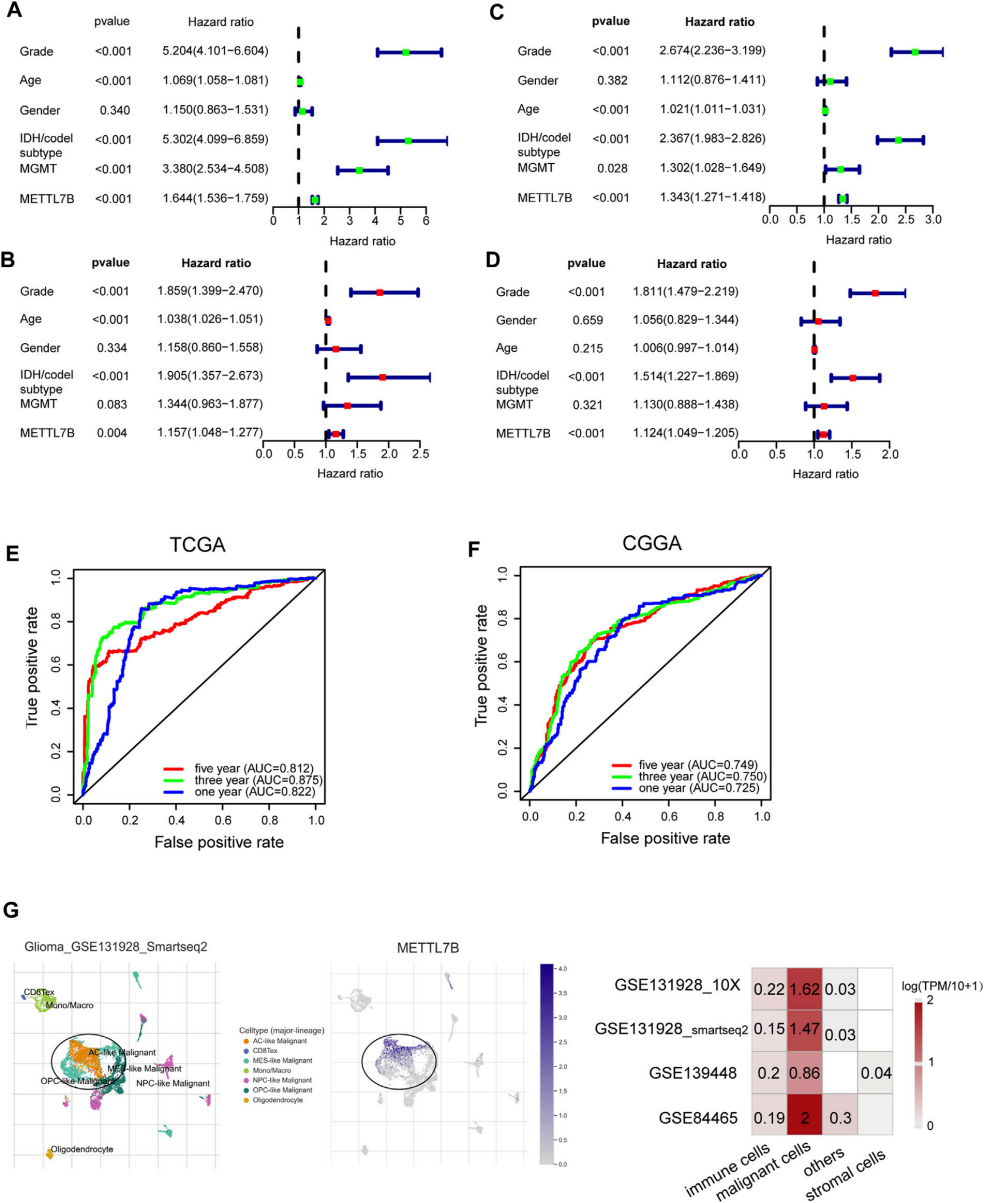
Relationship between METTL7B expression and prognosis of glioma patients. **(A–D)** Univariate and multivariate Cox analyses evaluating the independent prognostic value of METTL7B in terms of OS in glioma patients using TCGA and CGGA datasets. **(E–F)** Receiver operator characteristic curve analysis of METTL7B in TCGA and CGGA datasets. AUC, area under the curve. **(G)** Single-cell level analysis evaluating the expression of METTL7B.

Univariate Cox analysis showed that METTL7B expression level (HR = 1.644; 95% CI = 1.536–1.759; *p* < 0.001), grade, age, IDH/codel subtype, and MGMT promoter status were significantly correlated with poor OS (Figure 2A). Multivariate Cox analysis showed that high METTL7B expression level (HR = 1.157; 95% CI = 1.048–1.277; *p* = 0.004) in the TCGA database was independently associated with poorer OS. This finding implied that METTL7B was a potential independent prognostic indicator for glioma (Figure 2B). These findings were determined using data from the CGGA database (Figures 2C,D). Moreover, subgroup analysis in different WHO grade indicated that METTL7B was an independent predictor of poor prognosis (Supplementary Table S1–S2). In addition, METTL7B was a significant predictor of 1-year (AUC = 0.812), 3-year (AUC = 0.875), and 5-year survival (AUC = 0.812) using receiver operating characteristic curve analysis of TCGA data (Figure 2E). This finding was further validated using data retrieved from the CGGA database (Figure 2F). More interestingly, we found that the expression level of METTL7B was higher in malignant cells compared with immune cells and stromal cells in the GBM patients by using the TISCH database (Figure 2G).

### Relationship Between METTL7B Expression and Clinical-Pathological Features

**FIGURE 3:**
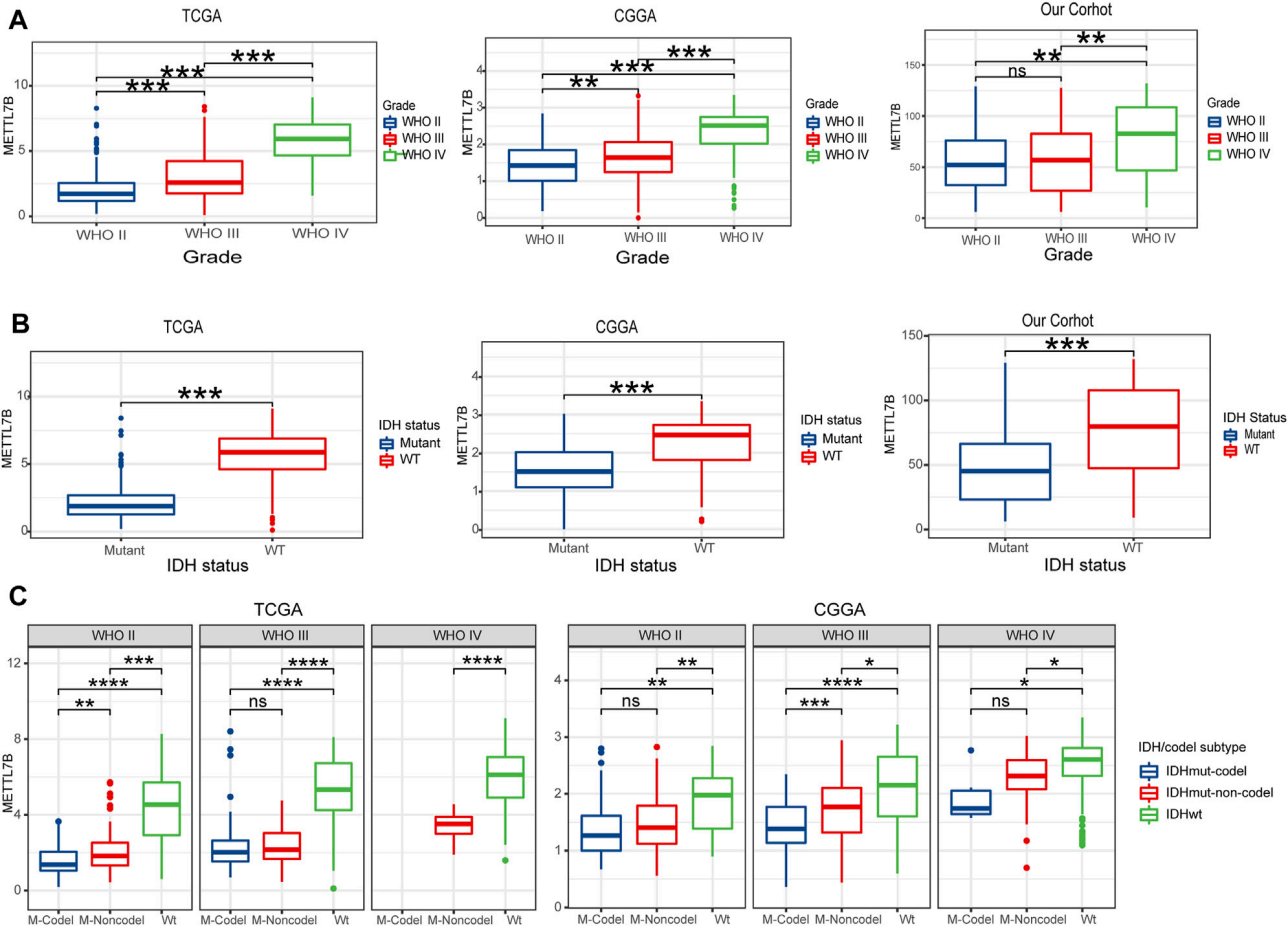
Relationship between METTL7B expression and clinical-pathological features. **(A)** Expression levels of METTL7B in gliomas with different WHO grades. **(B)** Expression levels of METTL7B in gliomas with different IDH status. **(C)** Expression levels of METTL7B with IDH/codel subtype in different WHO grades; *p < 0.05, **p < 0.01, ***p < 0.001, and ****p < 0.0001.

To determine the role of METTL7B in tumorigenesis and tumor development, we explored relationships between METTL7B and clinic-pathological features of gliomas, including WHO grade, IDH status, IDH/codel subtype. Glioma patients with high WHO grades, IDH-wild-type, showed significantly high expression of METTL7B. These correlations were confirmed using CGGA datasets and our datasets (Figures 3A,B). Moreover, subgroup analysis in different WHO grade indicated that the levels of METTL7B expression were higher in the IDHwt subtype compared with the IDHmut-non-codel and IDHmut-codel subtype (Figure 3C).

### Gene Set Enrichment Analysis of METTL7B Expression Data

GO and signaling pathways in low and high METTL7B expression groups were analyzed using gene set enrichment analysis using MSigDB Collection (c2.cp.kegg and c5.go.bp. v7.2. symbols) (FDR <0.05). Normalized enrichment score (NES) was used to determine the most significantly enriched GO and signaling pathways. In our study, negative regulation of immune effector process, negative regulation of T cell proliferation, negative regulation of B cell activation, negative regulation of CD4 positive alpha beta T cell activation, positive regulation of I kappab kinase NF kappab signaling, positive regulation of vasculature development, apoptosis, p53 signaling pathway, JAK STAT signaling pathway, cell adhesion molecules cams, and ECM receptor interaction pathways were enriched in METTL7B high expression phenotype (Supplementary Figure S1).

### Differences in DNA Methylation Related to METTL7B Expression

**FIGURE 4:**
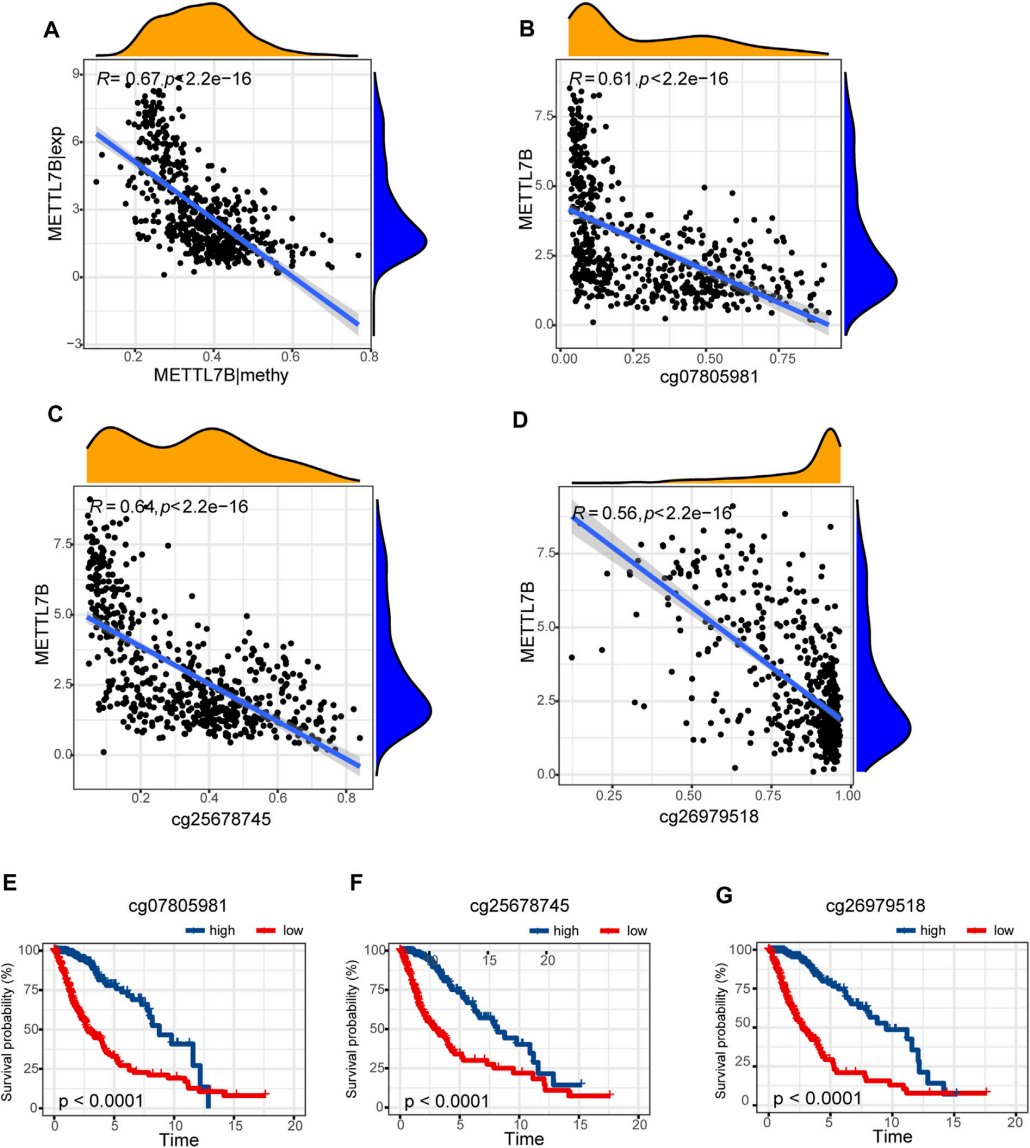
Differences in DNA methylation related to METTL7B expression. **(A)** Expression of METTL7B was negatively regulated by METTL7B DNA methylation. **(B–D)** Expression of METTL7B was significantly negatively regulated by METTL7B DNA promoter CpG sites, including cg07805981, cg25678745, and cg26979518. **(E–G)** Kaplan‐Meier analysis showed that high levels of these CpG sites were associated with good OS.

Hypermethylation in CpG islands and hypomethylation in CpG poor regions were defined as abnormal DNA methylation was positively correlated with enhanced tumorigenesis and tumor progression (Koch et al., 2018; Soozangar et al., 2018). Illumina Infinium 450k DNA methylation data from TCGA portal were used to identify and compare the effects of DNA methylation patterns in high and low METTL7B groups. METTL7B expression showed significant negative correlation (r = −0.65, *p* < 0.0001) with METTL7B DNA methylation (Figure 4A). The Pearson correlation analysis showed that methylation of METTL7B CpG sites was highly correlated with METTL7B expression. In addition, methylation of pall CpG sites, except for CpG sites cg05567435, negatively correlated with the expression of METTL7B (Table 1). Significant CpG sites (|r| > 0.5, *p* < 0.0001 (Figures 4B–D) were identified. Kaplan–Meier analysis was used to assess prognostic values of these significant METTL7B DNA CpG sites in patients with glioma. Analysis showed that high levels of these CpG sites were associated with good OS (Figures 4E–G).

**TABLE 1:** The methylation of CpGs sites negatively correlated with METTL7B.

Tag	*p* value	Cor
cg25678745	7.42E-73	−0.654
cg26979518	1.89E-69	−0.642
cg07805981	3.72E-48	−0.552
cg07530577	2.03E-26	−0.419
cg15702701	1.92E-10	−0.259
cg26537248	2.57E-10	−0.257
cg18610738	2.22E-05	−0.174
cg05567435	1.57E-01	−0.059

### Differences in Somatic Mutations Related to METTL7B Expression

**FIGURE 5:**
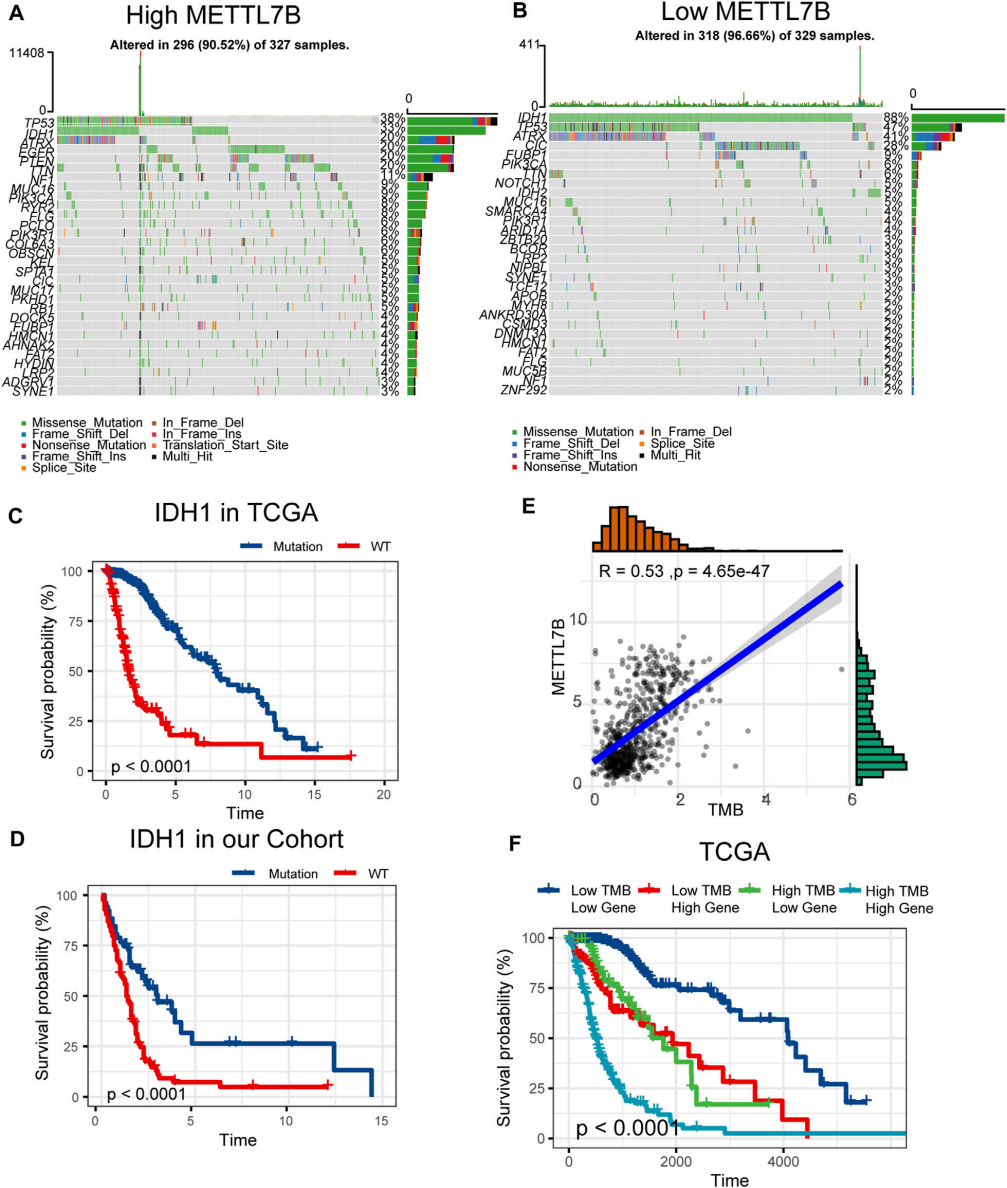
Differences in somatic mutations related to METTL7B expression. **(A–B)** Top 30 most frequently mutated genes between high and low METTL7B groups. **(C–D)** Survival analysis of gliomas with different IDH1 status in the TCGA dataset and our cohort. **(E)** Correlation curve between METTL7B and the level of TMB. **(F)** Survival analysis of gliomas between different TMB scores and high or low METTL7B expression levels.

Somatic mutations for high and low METTL7B expression cohorts were explored to find relevant genetic alterations. The top 30 most frequently mutated genes in the corresponding cohorts are shown in Figures 5A,B. Tumor driver genes (such as TTN, EGFR, and PTEN) showed high mutation rates in the high METTL7B cohort compared with the rates in the low METTL7B cohort. On the contrary, IDH1 mutation showed the highest mutation frequency in low METTL7B cohort compared with high METTL7B cohort. These differential mutation rates were validated using our dataset (Table 2). Our findings are consistent with reports from previous studies that IDH1 mutation are correlated with more favorable OS implying that IDH1 mutations play a critical role in glioma patients (Figures 5C, D). Moreover, top 10 genes with differential mutation frequencies between the two cohorts were identified using Fisher’s exact test (Table 2). Association between METTL7B expression and survival of gliomas patients with different TMB scores was further analyzed. We found that TMB exhibits a positive correlation with the level of METTL7B (Figure 5E). Samples in TCGA were divided into four groups based on TMB score and METTL7B expression level. The resulting groups were high TMB score with high or low METTL7B expression and low TMB score with high or low METTL7B expression. Analysis of these groups showed that low METTL7B with low TMB score group had a higher OS compared with high METTL7B group with high TMB score (Figure 5F).

**TABLE 2:** The most differential frequently mutated genes.

Gene	Low num	High num	*p* value
FUBP1	26	10	0.009
TP53	152	121	0.018
MUC16	15	29	0.029
IDH1	289	108	<0.0001
ATRX	125	62	<0.0001
CIC	89	16	<0.0001
TTN	20	64	<0.0001
PTEN	3	64	<0.0001
EGFR	1	64	<0.0001
NF1	6	32	<0.0001

### Estimation of Immune Cell-Type Fractions in Glioma

**FIGURE 6:**
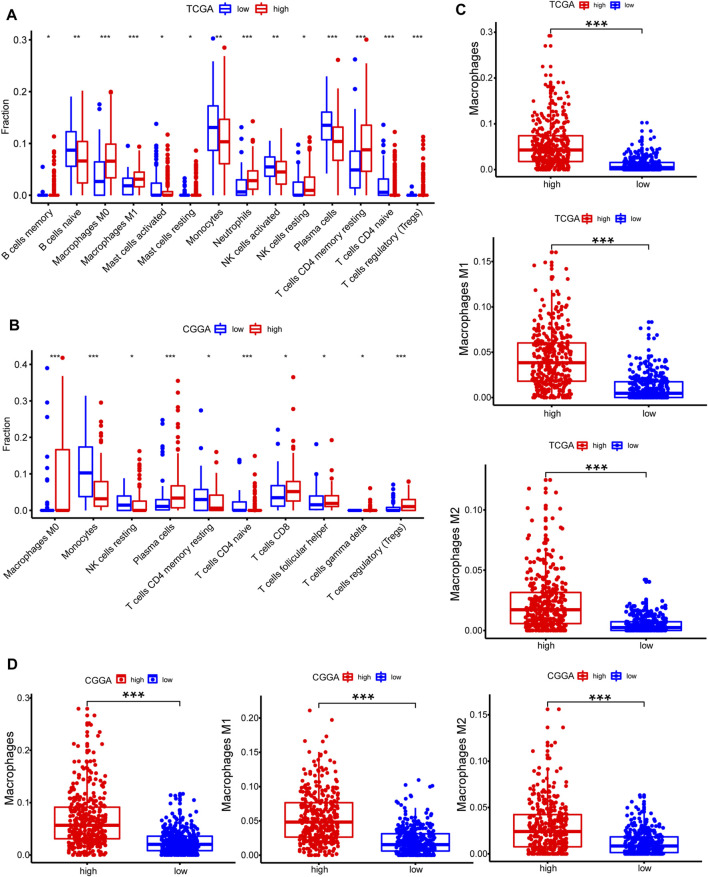
Estimation of immune cell-type fractions in glioma. **(A–B)** Difference in distribution of immune cell types in tumor microenvironment between low METTL7B and high METTL7B groups from 697 samples in TCGA and 693 samples in CGGA datasets as determined using CIBERSORT. **(C–D)** Analysis using xCell showed that TAMs were significantly higher in the high METTL7B expression group. *p < 0.05, **p < 0.01, ***p < 0.001, and ****p < 0.0001.

CIBERSORT method in combination with LM22 signature matrix and xCell (http://xcell.ucsf.edu/) was used to estimate differences in the distribution of immune cell types in the tumor microenvironment of low METTL7B and high METTL7B groups. Results obtained from 697 samples from TCGA and 693 samples from CGGA using CIBERSORT analysis are shown in Figures 6A,B. Patients with high METTL7B expression levels showed significantly higher expression levels of immunosuppressive cells (such as Tregs, TAMs, and neutrophils), NK cells, and rested T cells. On the contrary, patients with high METTL7B expression levels showed significantly lower levels of activated NK cells (Figure 6A). Similar to TCGA results, levels of TAMs and Tregs in the high METTL7B group were higher compared with levels in the low METTL7B group in the CGGA cohort (Figure 6B).

To further explore the potential function of immune cells in tumor infiltration, the expression model of TIICs in glioma with different METTL7B cohorts was studied using xCell (Supplementary Figure S1). Factors with a significant difference in infiltration ratio were selected. Analysis showed that TAMs were significantly higher in the low METTL7B expression group compared with the high METTL7B expression group (Figure 6C). TAMs originating from monocytes are an important type of immune cells in the tumor microenvironment, accounting for 50% of total immune cell counts. In addition, TAMs play an important role in neoplasia, metastasis, immune escape, and tumor angiogenesis (Liu and Cao 2015; de Groot and Pienta 2018). These findings imply that METTL7B plays an important role in the tumor immune microenvironment.

### High METTL7B Expression Indicates an Immunosuppressive Microenvironment

**FIGURE 7:**
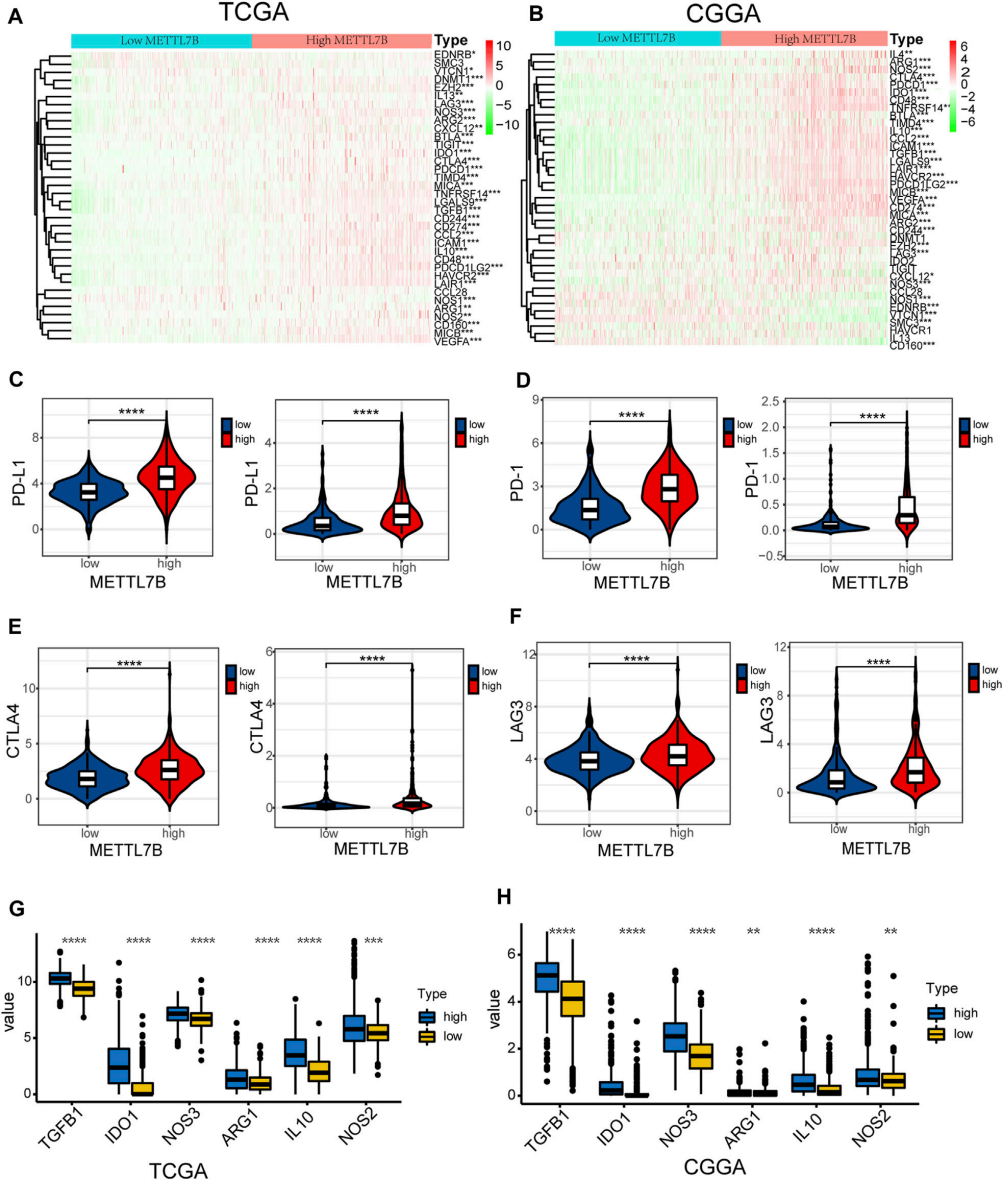
High METTL7B expression indicates an immunosuppressive microenvironment. **(A–B)** Heatmap of profiles of genes involved in negative regulation of the Cancer-Immunity Cycle in high and low METTL7B groups in the TCGA and CGGA datasets. **(C–F)** PD-L1, PD-1, CTLA4, and LAG3 expression levels in high and low METTL7B groups. **(G–H)** Tumor immunosuppressive cytokine expression in high and low METTL7B groups. *p < 0.05, **p < 0.01, ***p < 0.001, and ****p < 0.0001.

Cancer-Immunity Cycle provides a theoretical basis for cancer immunotherapy research (Chen and Mellman 2013). In this study, we explored the expression of genes negatively regulating The Cancer-Immunity Cycle in low and high METTL7B groups. The findings showed that these genes were mostly upregulated in the high METTL7B group (Figures 7A,B). This implies that patients in this group have lower activities of the immune microenvironment.

**FIGURE 8:**
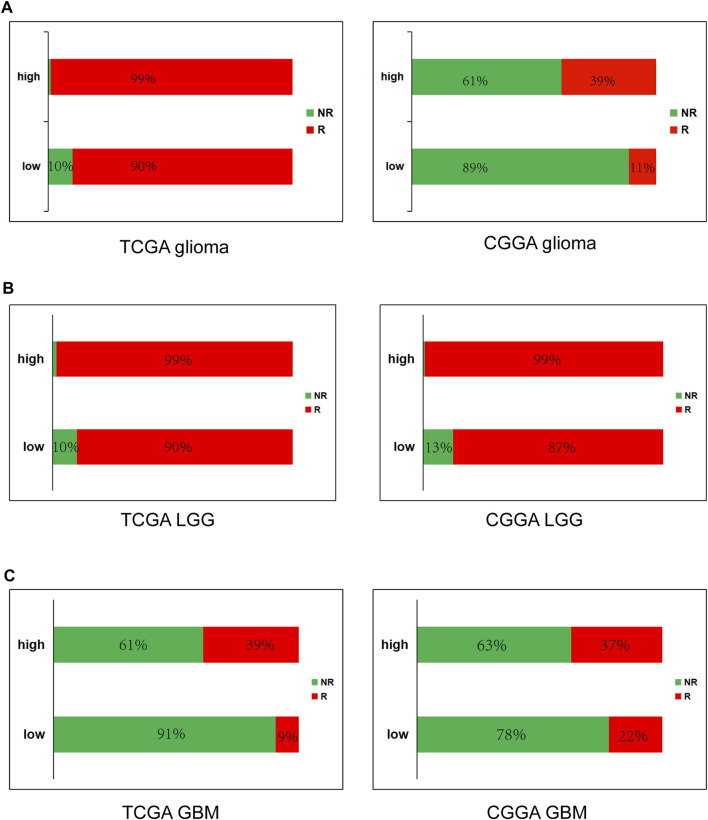
High METTL7B expression is correlated with response of ICB therapy as shown using ImmuCellAI in glioma **(A)**, LGG **(B)** and GBM **(C)**.

Moreover, we explored the relationship between several immune-associated molecules and the expression of METTL7B. PD1 and PD-L1 levels were high in the high METTL7B group compared with the levels in the low METTL7B group (Figures 7C,D). In addition, the expression of crucial immune checkpoints (i.e., CTLA-4 and LAG-3) in the high group was significantly higher compared with the levels in the low METTL7B group (Figures 7E,F). In addition, the expression levels of immunosuppressive cytokines were significantly higher in the high METTL7B group compared with levels in the low METTL7B group (Figures 8G,H). These findings imply that the high METTL7B expression level promotes the immunosuppressive microenvironment through upregulation of immune checkpoints and immunosuppressive cytokines.

### High METTL7B Predicts Response of Immune Checkpoint Blockade (ICB) Therapy

To further determine the effect of high METTL7B expression on immunotherapy of glioma patients, ImmuCellAI (http://bioinfo.life.hust.edu.cn/ImmuCellAI#!/), was used to predict immune checkpoint blockade (ICB) therapy response. Immune cell abundance estimated using TCGA and CGGA databases was used for the prediction. The results showed that glioma (LGG and GBM) patients with high expression levels of METTL7B have a better response to immunotherapy and GBM have worse response, compared to LGG (Figure 8).

### Construction and Validation of Prognosis Prediction Model

The above findings show significant METTL7B-related alterations in multi-omics characteristics including expression profile change, DNA methylation, somatic mutation, and immune cells. A total of 31 upregulated immunosuppressive genes and immune checkpoints were analyzed in the high METTL7B cohort in TCGA and CGGA datasets to explore expression changes. The top 10 frequently mutated genes were identified in the low METTL7B and high METTL7B cohorts to explore somatic mutations. Analysis of DNA methylation showed a total of 8 differential methylation probes at the regions of METTL7B. Moreover, a total of 21 differential immune cells were detected in differential METTL7B cohorts for the TCGA and CGGA datasets.

Univariate Cox regression analysis and LASSO-COX analysis were used to determine key prognostic biomarkers, and a risk signature was constructed. TCGA samples were randomly divided into training and independent test sets. Multi-omics data were missing from the other databases; therefore, they were not included in the analysis. The formula for the risk signature was determined using corresponding coefficients: risk signature = 0.1596 × (METTL7B) + 0.2345 × CD274 + 0.0149 × PDCD1 + −0.2305 × VTCN1 + 0.0026 × IL10 + 0.0823 × EZH2 + −0.7471 × cg07805981 + −0.3115 × cg25678745 + -0.6065 × cg26979518 + -0.3416 × Basophils (Xcell) + 0.0664 × CD4^+^ memory T cells (Xcell) + -0.1906 × eosinophils (Xcell) + -0.5024 × IDH1|snv + −0. 7,348 × CIC|snv.

**FIGURE 9:**
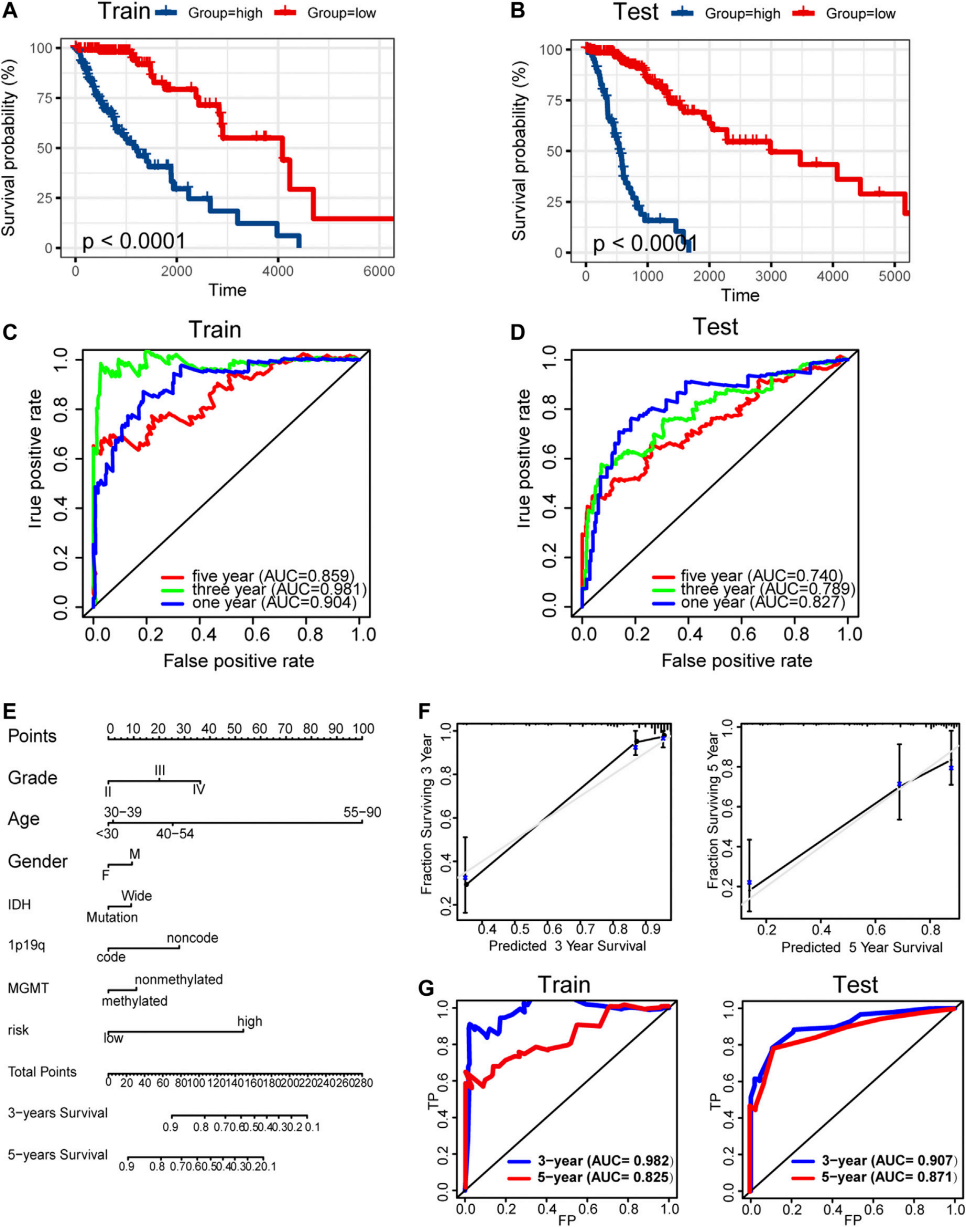
Construction and validation of prognosis prediction model. **(A–B)** Kaplan–Meier overall survival curves for gliomas assigned to high and low risk groups based on risk score in the training and test sets. **(C–D)** ROC curves showing predictive efficiency of the risk signature on 1-year, 3-year, and 5-year survival rate. **(E)** Nomogram for predicting 3- and 5-year overall survival with risk signature and clinical information in the training set. **(F)** Calibration curve of the nomogram using the training set. **(G)** ROC curves of the prediction index value in predicting 3- and 5-year overall survival of the training and test nomogram.

Kaplan–Meier analysis showed significantly poorer prognosis for glioma patients with high risk signature (*p* < 0.001, Figures 9A,B). ROC curve analysis showed that the risk signature had better-predictive power for 1-year, 3-year, and 5-year survival (AUC = 0.904.0.981.0.859 Figure 9C) compared with the use of METTL7B expression level (AUC = 0.781.0.841.0.812 Figure 2F) alone. This finding was further validated using the test set (Figure 9D). A prognostic nomogram was used as a quantitative tool to predict the survival of patients. The nomogram combined risk signature with clinical information of glioma patients (Figure 9E). Moreover, the calibration curve of the nomogram showed consistency between prediction and observation (C-index = 0.88) (Figure 9F). The training nomogram was not significantly different in 3-year (AUC = 0.982) and 5-year (AUC = 0.825) survival predictions (Figure 9G) compared with the risk signature constructed based on multi-omics alterations. However, the test nomogram displayed better-predictive power of 3- and 5-year survival (AUC = 0.907.0.871) compared with the risk signature constructed using the test set (Figure 9G).

## Discussion

This study presents the clinical prognostic value of METTL7B expression in glioma. Further, a significant association between METTL7B expression and clinical-pathological features is reported. Functional enrichment analysis showed that METTL7B negatively regulates immunity and carcinogenic signaling pathways. Moreover, a high METTL7B expression level is associated with high levels of immunosuppressive cells implying that it promotes the immunosuppressive microenvironment. A METTL7B-related prognostic signature was constructed and validated based on the multi-omics. The prognostic signature showed a high value in predicting the overall survival (OS) time of glioma patients. In conclusion, these findings provide insights on the pathologic role of METTL7B in promoting tumor progression and its potential value as a new diagnostic and prognostic biomarker for glioma.

METTL7B is an alkyl thiol methyltransferase that metabolizes hydrogen sulfide (H_2_S) (Maldonato et al., 2021). H_2_S is detected to induce NSCLC migration and invasion, as well as the epithelial-mesenchymal transition (EMT) process (Wang et al., 2020). In the advanced stage of thyroid cancer, METTL7B plays an important role in regulating EMT (Cai, Chen, Chen, Li, Du, and Zhou 2018). Moreover, METTL7B knockdown promoted cell cycle arrest at G0/G1phase and induced cellular apoptosis in clear cell renal cell carcinoma (Li et al., 2021). In our study, KEGG analysis demonstrated that METTL7B is involved in cell adhesion molecules cams and apoptosis. These findings imply that METTL7B may promote the growth and metastasis of glioma cells by being involved in the metabolism of H_2_S.

Integrative risk signature comprising immune-related alterations and epigenetic regulation was used to estimate the role of METTL7B due to the complex pathogenesis of glioma. Treg cells, TAMs, and neutrophils are the most abundant immunosuppressive cells in the tumor microenvironment. Treg cells are recruited into the tumor microenvironment (TME) and inhibit anti-tumor immune responses, thus affecting the effectiveness of cancer immunotherapy (Yano et al., 2019). TAMs are major components in TME. Monocytes undergo reprogramming into TAMs after recruitment at the tumor site leading to the gain of protumoral functions such as supporting tumor growth; promoting angiogenesis, tumor invasion, and metastases; and suppressing T cells responsible for antitumoral responses (Netea-Maier et al., 2018). High levels of immunosuppressive cells (Treg cells, TAMs, and neutrophils) were observed in the high METTL7B group. In addition, immunosuppressive cells were significantly enriched in negative regulation of immunity pathway indicating that METTL7B can be used to predict the immune microenvironment.

Tumor immunosuppressive cytokines and immune checkpoints play an important role in tumorigenesis and cancer development by enhancing tumor immune escape. Increased expression of tumor immunosuppressive cytokines is a key feature of immune cell exhaustion. TGF-β has been shown to suppress the immune response by inhibiting NK-cell activity, decreasing cytokine production, inhibiting dendritic cell maturation, and altering T-cell cytotoxic properties (Haque and Morris 2017). Previous studies report that M2-macrophages, Tregs, and Th2-cells produce IL-10, which is implicated in the impairment of proliferation, cytokine production, and migratory capacities of effector T cells (Barbi et al., 2014). Stimulation of immune checkpoint targets is the main mechanism through which tumors escape immune cells attack. In our study, immunosuppressive cytokines and immune checkpoints like PD1, PD-L1, LAG3, and CTLA-4 were upregulated in the high METTL7B group. These findings imply that METTL7B helps cancer cells in evading natural anti-tumor immune responses by decreasing the activity of NK or CTL cells, enhancing suppressive cells (Tregs, TAMs, and neutrophils), and increasing immunosuppressive molecular factors. Moreover, analysis using ImmuCellAI showed that patients with high METTL7B had a better response to immunotherapy. Therefore, targeting METTL7B may have significant clinical implications in improving immunotherapy.

A previous study reports that abnormal DNA methylation is implicated in the induction and progression of glioma (Mathur et al., 2020). In our study, METTL7B methylation showed a negative correlation with METTL7B mRNA expression in gliomas. Low methylation of METTL7B in glioma tissues is attributed to the negative correlation between METTL7B methylation and expression levels. We further explored specific CpG sites in METTL7B DNA promoter at which methylation is significantly correlated with METTL7B mRNA expression. Notably, cg07805981, cg25678745, and cg26979518 showed significant associations with METTL7B expression. Therefore, we evaluated the prognostic value of these three CpG sites. METTL7B hypermethylation showed a significantly high OS in patients with glioma. In summary, METTL7B expression was negatively regulated by METTL7B methylation, and METTL7B methylation status is a potential prognostic factor of OS.

Pathogenesis of tumor may be related to the variation of the exon group due to its complexity and involvement of multiple genes. Several genes, including IDH, TP53, PTEN, and EGFR, undergo mutations in gliomas (Wong et al., 1992; Cancer Genome Atlas Research Network, 2008). These mutations occur in a defined order during progression to a high-grade tumor. IDH mutations occur early in the development of glioma from a stem cell that gives rise to both astrocytes and oligodendrocytes (Yan et al., 2009). TP53 mutation is a relatively early event during the development of an astrocytoma, whereas loss or mutation of PTEN and amplification of EGFR are characteristics of higher-grade tumors (Weber et al., 1996; Furnari et al., 2007; Ohgaki and Kleihues 2007). In our study, tumors with IDH1 mutations showed a better outcome compared with those with wild-type IDH genes. Previous studies report that METTL7B can be induced by mutant P53 and wild-type P53 protein through interaction with the upstream promoter region of METTL7B (Neilsen et al., 2011). KEGG analysis in our study revealed that METTL7B is involved in p53 pathway. However, fewer p53 mutations were identified in the high METTL7B group which can be attributed to the complexity of high-grade gliomas. TMB, a novel biomarker for the prediction of immune responses, is effective in various cancers, such as breast cancer (Park et al., 2018; Thomas et al., 2018). In our study, the high METTL7B group with high TMB showed high OS compared with the low METTL7B group with low TMB. Similar results have been reported in most malignancies that higher TMB induces local immune recognition and improves prognosis.

Bioinformatics is a flourishing study approach. Through data analysis, many potential tumor markers can be found for the study of antitumor treatments. Previous studies have shown that m6A related genes may predict the prognosis or be used for the diagnosis of glioma (Dong and Cui 2020). Ye et al. found a novel ferroptosis-related gene signature for prognostic prediction in glioma patients and revealed the relationship between ferroptosis-related genes and immune checkpoint molecules (Chen et al., 2021). A hypoxia risk model based on five hypoxia-associated genes, which served as an independent prognostic indicator and reflected overall immune response intensity in the glioma microenvironment (Lin et al., 2020). The discovery of these biomarkers enables us to understand more about the mechanism of glioma development, thus aiding the clinical diagnosis and treatment of glioma. However, the prognosis of tumors is complex and cannot be predicted accurately by a single index. The combined analysis of multiple indicators or multi-omics will improve the accuracy of prognosis prediction. In the present study, a risk signature constructed based on multi-omics characteristics showed a superior prediction capability with a higher AUC value compared with the risk signature constructed from METTL7B levels alone. In addition, a comprehensive nomogram, in which combined risk signature was built using clinical information of glioma patients, has a favorable advantage in prognostic prediction.

Although this study provides a more integrative view of the association between METTLL7B expression and glioma and a prognostic model with high predictive capabilities, it has limitations that should be explored further. Notably, matching multi-omics data was missing in other data sources. This prevented us from examining the robustness of the model when used for other data.

In summary, our findings revealed that METTL7B was overexpressed in glioma and identified it as a promising prognostic biomarker. Moreover, the METTL7B expression level was positively correlated with immunosuppressive cells implying that it may play an important role in regulating the microenvironment. Our prognostic prediction model can be used in clinical applications to improve OS and for the development of new effective therapeutic strategies for glioma patients.

## Data Availability

The original contributions presented in the study are included in the article/Supplementary Material; further inquiries can be directed to the corresponding authors.

## Abbreviations

ALD1, associated with lipid droplets; CGGA, Chinese glioma genome atlas; EMT, epithelial-mesenchymal transition; FDR, false discovery rate; GBM, glioblastoma; GEPIA, gene expression profiling interactive analysis; GSEA, gene set enrichment analysis; GO, gene ontology; H-score, histochemistry score; IDH, isocitrate dehydrogenase; IHC, immunohistochemistry; LGG, low-grade glioma; METTL7B, methyltransferase-like 7B; NES, normalized enrichment score; OS, overall survival; ROC, receiver operating characteristic; TAM, tumor-associated macrophage; TMB, tumor mutation burden; TME, tumor immune microenvironment; WES, whole exome sequencing; TCGA, The Cancer Genome Atlas.
