# Supplementary figures and images for: Characterization of METTL7B to Evaluate TME and Predict Prognosis by Integrative Analysis of Multi-Omics Data in Glioma
Source: Front Mol Biosci. 2021 Sep 17;8:727481. doi: 10.3389/fmolb.2021.727481 (PMC8484875; doi:10.3389/fmolb.2021.727481)

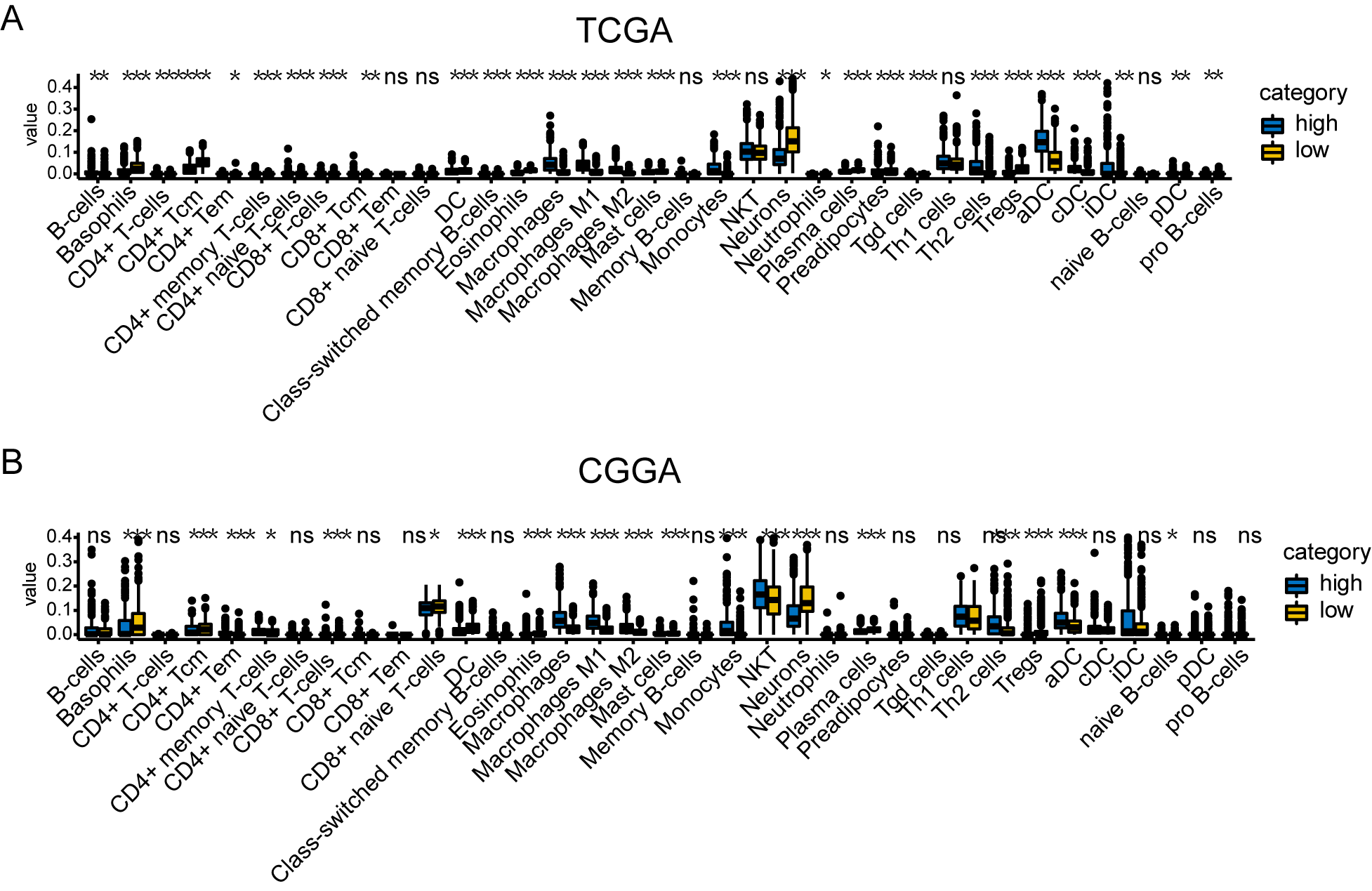

Supplement: Supplementary file 1 [file Image2.TIF]

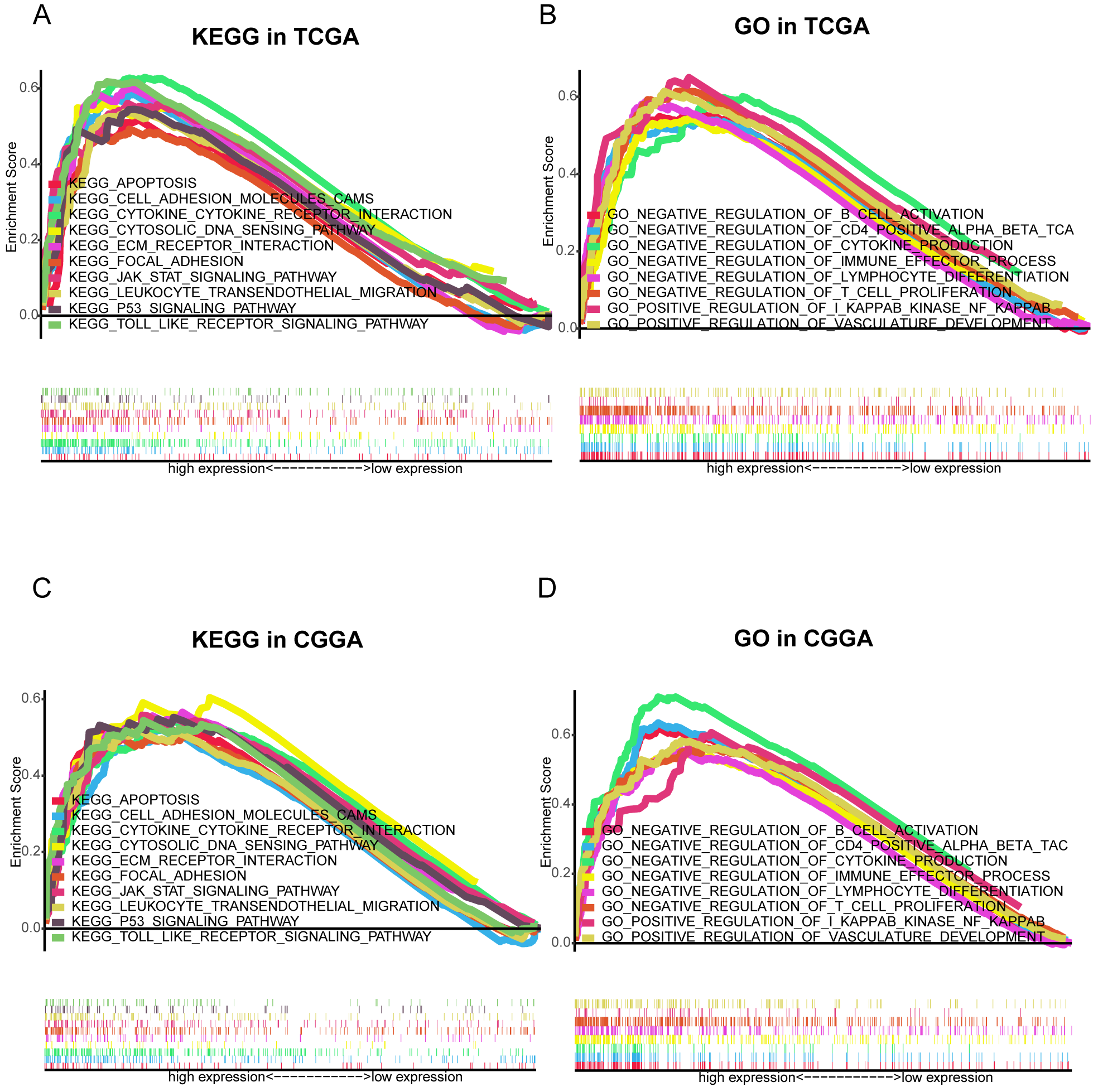

Supplement: Supplementary file 2 [file Image1.TIF]
