# Supplementary material for: Characterization of METTL7B to Evaluate TME and Predict Prognosis by Integrative Analysis of Multi-Omics Data in Glioma
Source: Front Mol Biosci. 2021 Sep 17;8:727481. doi: 10.3389/fmolb.2021.727481 (PMC8484875; doi:10.3389/fmolb.2021.727481)
Supplement: Supplementary file 4 [file DataSheet1.PDF]

Tab.S1. Multivariate analysis of the correlation of METTL7B expression with OS in TCGA

		HR	HR.95L	HR.95H	pvalue
WHO II					
	Gender	1.013	0.463	2.219	0.973
	Age	1.040	1.009	1.071	0.010
	IDH/codel subtype	2.856	1.169	6.979	0.021
	MGMT	0.801	0.249	2.578	0.710
	METTL7B	1.308	1.005	1.702	0.046
WHO III					
	Gender	1.143	0.708	1.843	0.585
	Age	1.056	1.035	1.079	<0.001
	IDH/codel subtype	1.891	1.193	2.996	0.007
	MGMT	1.243	0.689	2.244	0.470
	METTL7B	1.220	1.041	1.429	0.014
WHO IV					
	Gender	1.036	0.653	1.644	0.880
	Age	1.021	1.001	1.041	0.041
	IDH/codel subtype	3.116	0.788	12.320	0.105
	MGMT	1.627	1.026	2.579	0.038
	METTL7B	1.080	0.996	1.148	0.080

Tab.S2. Multivariate analysis of the correlation of METTL7B expression with OS in CGGA

		HR	HR.95L	HR.95H	pvalue
WHO II					
	Gender	1.094	0.528	2.268	0.808
	Age	1.028	0.995	1.062	0.101
	IDH/codel subtype	1.261	0.748	2.124	0.384
	MGMT	1.863	0.904	3.842	0.092
	METTL7B	1.354	1.071	1.702	0.011
WHO III					
	Gender	1.842	1.216	2.788	0.004
	Age	1.000	0.983	1.018	0.976
	IDH/codel subtype	1.967	1.377	2.809	<0.001
	MGMT	0.990	0.647	1.513	0.962
	METTL7B	1.176	1.059	1.305	0.002
WHO IV					
	Gender	0.805	0.571	1.135	0.216
	Age	1.007	0.995	1.018	0.260
	IDH/codel subtype	1.545	1.077	2.216	0.018
	MGMT	1.120	0.797	1.573	0.514
	METTL7B	1.136	1.002	1.296	0.023
